# End-to-End Thiocyanato-Bridged Helical Chain Polymer and Dichlorido-Bridged Copper(II) Complexes with a Hydrazone Ligand: Synthesis, Characterisation by Electron Paramagnetic Resonance and Variable-Temperature Magnetic Studies, and Inhibitory Effects on Human Colorectal Carcinoma Cells

**DOI:** 10.1002/open.201100011

**Published:** 2012-03-13

**Authors:** Kuheli Das, Amitabha Datta, Chittaranjan Sinha, Jui-Hsien Huang, Eugenio Garribba, Ching-Sheng Hsiao, Chin-Lin Hsu

**Affiliations:** [a] Department of Chemistry, Jadavpur University188 Raja S. C. Mallik Road, 700032 Kolkata (India); [b] Department of Chemistry, National Changhua University of Education1 Jin-De Road, 50058 Changhua (Taiwan) E-mail: amitd_ju@yahoo.co.injuihuang@cc.ncue.edu.tw; [c] Dipartimento di Chimica and Centro Interdisciplinare per lo Sviluppo della Ricerca Biotecnologica e per lo Studio della Biodiversità della Sardegna, Università degli Studi di SassariVia Vienna 2, 07100 Sassari (Italy); [d] School of Nutrition, Chung Shan Medical UniversityNo. 110, Sec.1, Cheng-Kuo N. Rd., 40201 Taichung (Taiwan)Department of Nutrition, Chung Shan Medical University HospitalNo. 110, Sec.1, Jianguo N. Rd., 40201 Taichung (Taiwan)

**Keywords:** copper(II) complexes, electron paramagnetic resonance spectroscopy, hydrazone ligands, variable-temperature magnetic studies

## Abstract

The reactions of the tridentate hydrazone ligand, *N*′-[1-(pyridin-2-yl)ethylidene]acetohydrazide (HL), obtained by condensation of 2-acetylpyridine with acetic hyadrazide, with copper nitrate trihydrate in the presence of thiocyanate, or with CuCl_2_ produce two distinct coordination compounds, namely a one-dimensional helical coordination chain of [CuL(NCS)]_*n*_ (**1**) units, and a doubly chlorido-bridged dinuclear complex [Cu_2_L_2_Cl_2_] (**2**) (where L=CH_3_C(O)=N–N=CCH_3_C_5_H_4_N). Single-crystal X-ray structural determination studies reveal that in complex **1**, a deprotonated hydrazone ligand L^−^ coordinates a copper(II) ion that is bridged to two neighbouring metal centres by SCN^−^ anions, generating a one-dimensional helical coordination chain. In complex **2**, two symmetry-related, adjacent copper(II) coordination entities are doubly chlorido-bridged, producing a dicopper entity with a Cu⋅⋅⋅Cu distance of 3.402 (1) Å. The two coordination compounds have been fully characterised by elemental analysis, spectroscopic techniques including IR, UV–vis and electron paramagnetic resonance, and variable-temperature magnetic studies. The biological effects of **1** and **2** on the viability of human colorectal carcinoma cells (COLO-205 and HT-29) were evaluated using an MTT assay, and the results indicate that these complexes induce a decrease in cell-population growth of human colorectal carcinoma cells with apoptosis.

## Introduction

Arylhydrazone-based coordination compounds of transition-metal ions have been used for the elucidation of the mechanism of enzyme inhibition by hydrazine derivatives,[1] and for their possible pharmacological applications.[2] Additionally, hydrazone-containing complexes have been the subject of numerous studies for many years due to their potential antimicrobial and antitumor activities.[3] As a result of their keto–enol tautomerism, they can act as neutral,[4] mono-,[5] di-,[6] or tetra-anionic[7] ligands, and, therefore, exhibit a highly versatile coordination behaviour towards cations.6a, [8] Hence, they represent a valuable class of ligands to generate interesting molecular architectures and coordination polymers. Some of their transition-metal complexes show electrical and magnetic properties,[9] or are used in synthetic and analytical chemistry as heterogeneous catalysts in redox processes, various chemical and photochemical reactions,[10] as well as in numerous industrial applications. Coordination compounds with arylhydrazone ligands are of great interest as well, since they can have attractive biological applications.[3, 11] In that context, copper(II) hydrazone compounds with remarkable biological activities, for example as anticancer agents, have been reported.[12] In addition, hydrazone-based copper complexes can be mentioned for their structural richness, as well as for their use to study the effects of structural and chemical factors that govern the exchange coupling between paramagnetic centres.[13] The design and preparation of pseudohalide-bridged dinuclear and polynuclear complexes remains an attractive area of investigation, since compounds with diverse architectures can be obtained with potential applications as magnetic materials.[14] The magnetic interactions between the metal centres of such complexes can be tuned by adjusting the chain length and bite angle of the bridging ligands. For instance, anions such as thiocyanates play an important role in determining the structure of polymeric transition-metal complexes.[15]

**Scheme 1:**
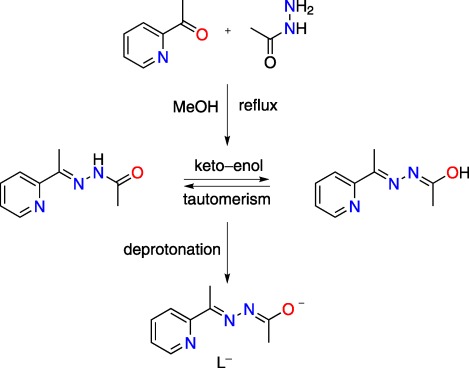
Condensation of acetic hydrazide with 2-acetylpyridine. A subsequent deprotonation leads to the formation of L^−^.[17]

Coordination compounds from aromatic hydrazones have been thoroughly investigated, including by some of us,[16] in contrast, metal complexes based on aliphatic hydrazones have yet to be explored. Herein, we describe two distinct copper(II) complexes: a one-dimensional helical coordination chain of [CuL(NCS)] (**1**) units and a doubly chlorido-bridged dinuclear complex [Cu_2_L_2_Cl_2_] (**2**), where L symbolises the deprotonated form of the ligand *N*′-[1-(pyridin-2-yl)ethylidene]acetohydrazide (HL). The ligand HL was obtained from an aliphatic hydrazone ligand as described previously by condensation of acetic hydrazide with 2-acetylpyridine (Scheme 1).[17] The present paper describes the synthesis and full characterisation of these two new copper(II) complexes **1** and **2** by IR, UV–vis and electron paramagnetic resonance (EPR) spectroscopies, single-crystal X-ray diffraction analysis and variable-temperature magnetic susceptibility studies. Moreover, the potential anticancer properties of **1** and **2** have been examined through cell-growth inhibition assays using human colorectal carcinoma cells (COLO-205 and HT-29).

## Results and Discussion

### Crystal structures

For both complexes **1** and **2**, details of the structure solution and refinement are summarised in Table 1 and selected bond distances and angles are listed in Table 2.

**Table 1:** Crystallographic data for complexes 1 and 2

	Complex **1**	Complex **2**
Empirical formula	C_10_H_10_CuN_4_OS	C_18_H_20_Cl_2_Cu_2_N_6_O_2_
Formula weight	297.84	550.38
*T* [K]	298(2)	150(2)
Wavelength [Å]	0.71073	0.71073
Crystal system	monoclinic	monoclinic
Space group	*P*2_1_/*n*	*C*2/*c*
Unit cell dimensions [Å]	*a*=12.4856(10)	*a*=14.032(3)
	*b*=7.5314(6)	*b*=9.1084(18)
	*c*=12.7061(10)	*c*=17.128(4)
Angle (*β*) [°]	98.516(4)	109.763(11)
Volume [Å^3^]	1181.63(16)	2060.1(7)
*Z*	4	4
Calcd density [mg m^−3^]	1.674	1.775
Absorption coefficient [mm^−1^]	2.012	2.353
*F*(000)	604	1112
Crystal size [mm^3^]	0.40×0.21×0.15	0.18×0.16×0.14
θ range for data collection [°]	2.13–28.78	2.53–28.73
Reflections collected	17 434	7940
Independent reflections	3056 [*R*(int)=0.0231]	2426 [*R*(int)=0.0453]
Data/restraints/parameters	3056/0/147	2426/0/138
Goodness-of-fit on *F*^*2*^	1.031	1.069
Final *R* indices [*I>2σ(I)*]	*R*1=0.0248	*R*1=0.0334
	*wR*2=0.0679	*wR*2=0.0650
*R* indices (all data)	*R*1=0.0293	*R*1=0.0848
	*wR*2=0.0705	*wR*2=0.0758
Largest diff. peak/hole [e Å^−3^]	0.533/−0.421	0.777/−1.188

**Table 2:** Selected bond lengths [Å] and angles [°] for complexes 1 and 2

Complex **1**[a]		Complex **2**[b]	
Cu1–O1	1.960(1)	Cu1–Cl1	2.746(1)
Cu1–N1	2.021(1)	Cu1–N2	1.923(2)
Cu1–N2	1.920(1)	Cu1–O1	1.963(2)
Cu1–N4	1.916(1)	Cu1–N3	2.023(2)
N2–N3	1.377(2)	Cu1–Cl1a	2.241(1)
Cu1–S1b	2.745(1)	N1–N2	1.382(3)
Shortest Cu⋅⋅⋅Cu	6.116(1)	Cu1⋅⋅⋅Cu1a	3.402(1)
N1–Cu1–N2	80.36(5)	N3–Cu1–N2	80.20(7)
N2–Cu1–O1	79.92(5)	O1–Cu1–Cl1a	99.49(5)
O1–Cu1–N4	98.56(5)	N3–Cu1–Cl1a	99.04(5)
N4–Cu1–N1	99.21(5)	N2–Cu1–O1	79.88(7)
		O1–Cu1–N3	159.35(8)
		N2–Cu1–Cl1a	169.10(6)

[a] Symmetry operation: *b*=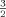 −*x*, 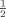 +*y*, 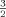 −*z*.

[b] Symmetry operation: *a*=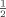 −*x*, 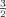 −*y*, −z.

#### [CuL(NCS)]_*n*_ (1)

**Figure 1:**
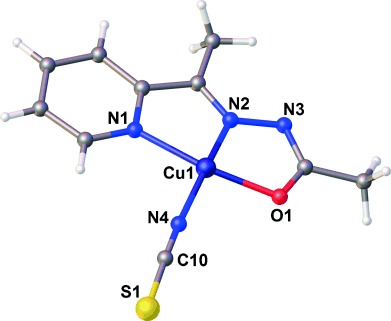
Representation of the molecular structure of [CuL(NCS)] (1).

**Figure 2:**
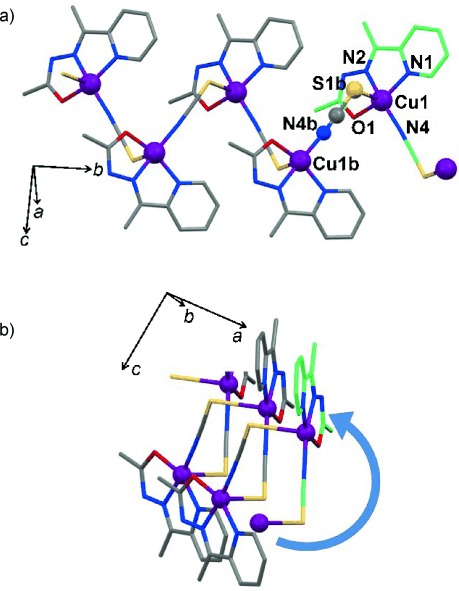
a) Crystallographic packing of complex 1 showing the S coordination of a thiocyanate at the axial position of a square-pyramidal copper(II) ion (Cu1–S1*b*=2.745(1) Å), generating a one-dimensional coordination polymer; b) the NCS bridging of molecules of 1 produces a one-dimensional helical chain that grows along the crystallographic *b* axis. Hydrogen atoms are omitted for clarity and the coordination compound is shown in green. Symmetry operation: *b*=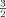 −*x*, 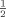 +*y*, 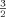 −*z*.

Reaction of copper(II) nitrate trihydrate with *N*′-[1-(pyridin-2-yl)ethylidene]acetohydrazide (HL) in methanol in the presence of sodium thiocyanate produces the mononuclear coordination compound [CuL(NCS)] (**1**). Single-crystal X-ray studies revealed that **1** crystallises in the monoclinic space group *P*2_1_/*n*. A view of the molecular structure of **1** is represented in Figure 1. The copper(II) ion appears to exhibit a square-planar geometry. However, the metal centre is semi-coordinated by a sulfur atom (Cu1–S1: *b*=2.745(1); Table 2) from a thiocyanate anion bound to a neighbouring molecule of **1**, which generates a five-coordinate copper(II) ion displaying a distorted square-pyramidal environment, whose basal plane contains the atoms O1, N1, N2 and N4 (Figure 1 and Figure 2 a). The distortion of the coordination polyhedron from square pyramidal to trigonal bipyramidal is expressed as *τ*, an index of the degree of trigonality; *τ* is defined as (β−α)/60, where β and α are the two *trans*-basal angles, and is 0.00 for a square pyramid and 1.00 for a trigonal bipyramid.[18] The value of *τ* for complex **1** (*τ*=0.20) is most likely caused by the small bite angles of the tridentate N,N,O-ligand (N1–Cu1–N2=80.36(5)° and N2–Cu1–O1=79.92(5)°; Table 2). The Cu–N, Cu–O and Cu–S bond lengths are in normal ranges for this type of CuN_3_OS chromophore.[19] The bridging thiocyanate anions connect each copper(II) ion to two neighbouring ones (the Cu⋅⋅⋅Cu separation distance is 6.116(1) Å; Table 2), generating a one-dimensional coordination polymer (Figure 2). This helical one-dimensional chain grows along the crystallographic *b* axis (Figure 2 b). The shortest interchain separation in the *c* direction is 8.831(1) Å.

#### [Cu_2_L_2_Cl_2_] (2)

**Figure 3:**
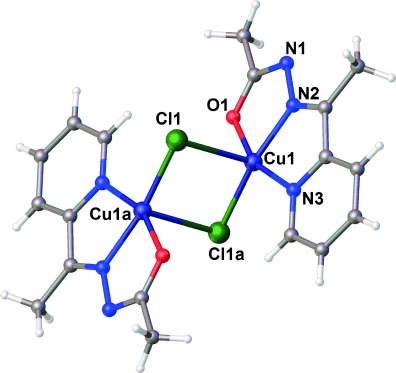
Representation of the molecular structure of compound 2. Symmetry operation: *a*=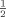 −*x*, 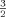 −*y*, −z.

**Figure 4:**
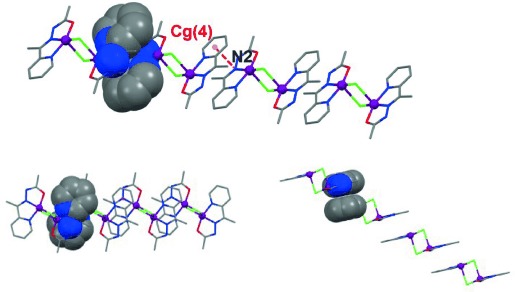
Different perspectives of the crystal packing of compound 2 showing the formation a staircase-like supramolecular chain generated by means of N(lone pair)–π interactions. The Cg4⋅⋅⋅N2 contact distance amounts to 3.421(2) Å.

The reaction of copper(II) chloride with *N*′-[1-(pyridin-2-yl)ethylidene]acetohydrazide (HL) in methanol yields the dinuclear coordination compound [Cu_2_L_2_Cl_2_] (**2**). Single-crystal X-ray studies revealed that **2** crystallises in the monoclinic space group *C*2/*c* (Table 1). A representation of the molecular structure of compound **2** is depicted in Figure 3, and selected bond lengths and angles are listed in Table 2. The structure determination shows that the coordination compound is a centrosymmetric dimer in which the metal ions are five coordinated. The copper(II) centre is coordinated to two N atoms and one O atom from a deprotonated tridentate L^−^ unit, which acts as a chelating, planar ligand, as observed for compound **1**. The remaining positions are occupied by two bridging chloride anions (Figure 3). The copper atoms Cu1 and Cu1a adopt a distorted square-pyramidal geometry characterised by a *τ* value of 0.16. The basal plane of the square pyramid is formed by the planar N,N,O-ligand and a chloride ion (atoms O1, N2, N3 and Cl1a; Figure 3). The elongated apical position is occupied by a second chloride anion Cu(1)–Cl(1)=2.746(1) Å; Table 2). The apical metal–halogen bond distance is longer (Cu–Cl1=2.746(1) Å) than the equatorial metal–halogen one (Cu–Cl1a=2.241(1) Å), as observed for related copper compounds reported earlier.[20] Complex **2** exhibits a dicopper(II) unit with a nearly rectangular Cu_2_(μ-Cl)_2_ core (angles: Cu1–Cl1–Cu1a=85.41(2)°; Cl1–Cu1–Cl1a=94.59(2)°), similar to that of compounds described in the literature, such as the dimethylenediamine (dmen)-derived copper complex [Cu_2_Cl_2_(dmen)_2_],[21] or the 3-methyl-2-(((pyridin-2-yl)methylene)amino)phenolato (3-Me-pyp) copper complex [Cu(3-Me-pyp)Cl].[22] The double chloride bridge gives rise to a Cu⋅⋅⋅Cu separation distance of 3.402(1) Å within the dimeric moiety, which displays an inversion centre located halfway between the copper(II) ions. The crystal packing of **2** shows the presence of staircase-like supramolecular chains that are generated by means of N(lone pair)–π interactions (Figure 4).[23] These supramolecular contacts are characterised by a centroid Cg4⋅⋅⋅N2 distance of 3.421(2) Å.[24]

### IR and electronic spectra

The IR spectra (range 4000–200 cm^−1^) of complexes **1** and **2** display absorption bands at 1592 and 1602 cm^−1^, respectively, which are assigned to the C=N stretching frequencies of the coordinated ligand; for the free ligand this band is observed at 1658 cm^−1^. The shifts of this band towards lower wave numbers upon coordination are indicative of the binding of the azomethine nitrogen atom to the metal centre.[25] The IR spectrum of the free hydrazone molecule contains a strong C–O absorption band at 1651–1659 cm^−1^. For the coordination compounds, this band is not present; instead, a new C–O absorption peak appears at 1198 and 1177 cm^−1^ for complexes **1** and **2**, respectively, which clearly suggests that HL undergoes deprotonation to L^−^ upon coordination. The coordination of the anion L^−^ to the copper(II) ion is substantiated further by prominent bands observed at 445 and 370 cm^−1^ for **1**, and at 437 and 378 cm^−1^ for **2**, which can be attributed to the *ν*_Cu–N_ and *ν*_Cu–O_ vibrations, respectively. A strong band is observed at 2133 cm^−1^ for **1**, which characterises the presence of bridging SCN^−^ anions. The two bands observed at 310 and 297 cm^−1^ for **2** are due to vibrations of the Cu–Cl bonds.[26] Strong, well-resolved, sharp absorption bands are found in the region 1495–1063 cm^−1^ for **1**, and 1488–1130 cm^−1^ for **2**, which are both assigned to coordinated pyridine rings.[25]

The electronic spectral data for both coordination compounds, recorded in HPLC grade acetonitrile, are in good agreement with their geometry. The UV absorption bands observed in the range of 217 and 281 nm for **1**, and 223 and 298 nm for **2** are due to π–π* transition within the hydrazine ligand. The UV absorption band observed at 385 for **1**, and 370 nm for **2**, is ascribed to the ligand–metal charge-transfer transition (LMCT) between the hydrazone ligand and cop per(II).[27, 28] The visible region of the spectrum for **1** displays a single broad band between 525 and 640 nm. These spectral features are consistent with the five-coordinate geometry of **1**. Typically, copper(II) coordination compounds with a square-pyramidal or distorted square-pyramidal geometry exhibit a band in the range 550–660 nm, whereas trigonal-bipyramidal complexes usually show a maximum at a *λ* value greater than 800 nm, which is associated with a high-energy shoulder.[29]

### EPR spectroscopy and temperature-dependant magnetic susceptibility measurements

**Figure 5:**
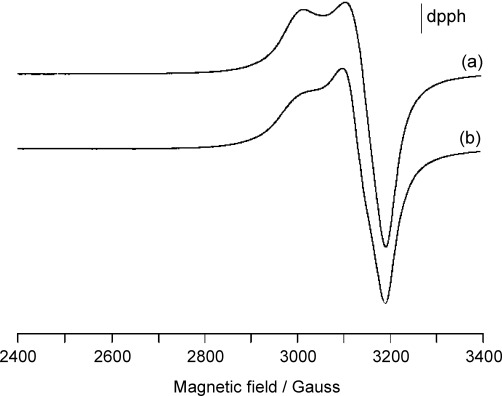
X-band EPR spectrum of a polycrystalline sample of complex 1 at RT: a) experimental and b) simulated spectrum.

**Figure 6:**
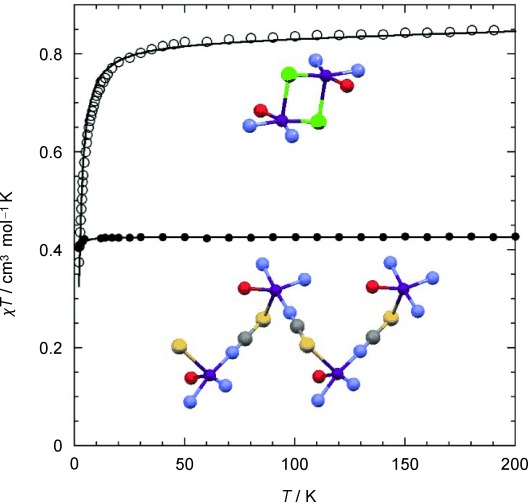
A plot of *χ*_M_ *T* versus *T* for complexes 1 (•) and 2 (○), where *χ*_m_ is the molar magnetic susceptibility. Only data below 200 K are shown. Lines represent the best fits to the adequate model (see text).

The EPR spectra of polycrystalline samples of **1** recorded at room temperature and 100 K are characterised by a slight rhombicity and three *g* values (*g*_x_=2.174, *g*_y_=2.074, *g*_z_=2.050 at RT, and *g*_x_=2.172, *g*_y_=2.078, *g*_z_=2.050 at 100 K) with the order *g*_x_>*g*_y_>*g*_z_>*g*_e_.[30] The experimental and simulated spectrum at room temperature are shown in Figure 5 (for experimental and simulated spectrum at 100 K, see Figure S1 in the Supporting Information). No resonances below 2500 and above 3500 G are detected. In these situations, the ground state can be described as a linear combination of *d*_*x*^2^−*y*^2^_ and *d*_*z*^2^_ orbitals,[31] and the parameter *R*=(*g*_y_−*g*_z_)/(*g*_x_−*g*_y_) is indicative of the predominance of *d*_*x*^2^−*y*^2^_ or *d*_*z*^2^_ orbital (if *R*>1, the greater contribution to the ground state arises from *d*_*z*^2^_; if *R* <1, the greater contribution to the ground state arises from *d*_*x*^2^−*y*^2^_ orbital).[30] The *R* values for **1** (0.24 at RT and 0.30 at 100 K) confirm the distorted square-pyramidal arrangement, for which a ground state based on the *d*_*x*^2^−*y*^2^_ orbital is expected.[30] The *χ*_m_ *T* product (molar magnetic susceptibility: *χ*_m_) of **1** remains practically constant at approximately 0.422–0.427 cm^3^ mol^−1^ K from 300 down to 4.5 K and only then decreases very slightly down to 0.404 cm^3^ mol^−1^ K at 2 K (see Figure 6), indicating the presence of very weak antiferromagnetic interactions. Given the thiocyanato-bridged, one-dimensional arrangement of the copper(II) ions in the structure of **1**, the experimental susceptibility data were fitted to the Bonner and Fisher regular chain model,[32] providing a good simulation (solid line in Figure 6) for the best-fit parameters: *g*=2.134(1) and *J*/*k*_B_=−0.14(1) K (or *J*=0.10(1) cm^−1^). The *g* value is in good agreement with the EPR data, while the very weak antiferromagnetic coupling can be satisfactorily correlated with the structural parameters of the thiocyanate bridge in **1**. Its end-to-end coordination mode, occupying an equatorial position at one copper(II) ion and an apical position at the adjacent one, with a long axial Cu–S bond length of 2.745(1) Å, can only result in a very weak exchange coupling, given the absence of spin density at the apical position of the copper(II) ion in a square-pyramidal environment. Indeed, similarly weak exchange couplings, with |*J*| values ranging up to 0.1 cm^−1^ have been reported for structurally characterised, single thiocyanato-bridged copper(II) complexes, for which the arrangement of the donor atoms around the metal centre is square pyramidal.14b, [33] The low efficiency of the thiocyanato ligand to mediate magnetic interactions through the out-of-plane pathway contrasts with that observed when it binds to the equatorial positions of two adjacent copper(II) ions.[34]

**Figure 7:**
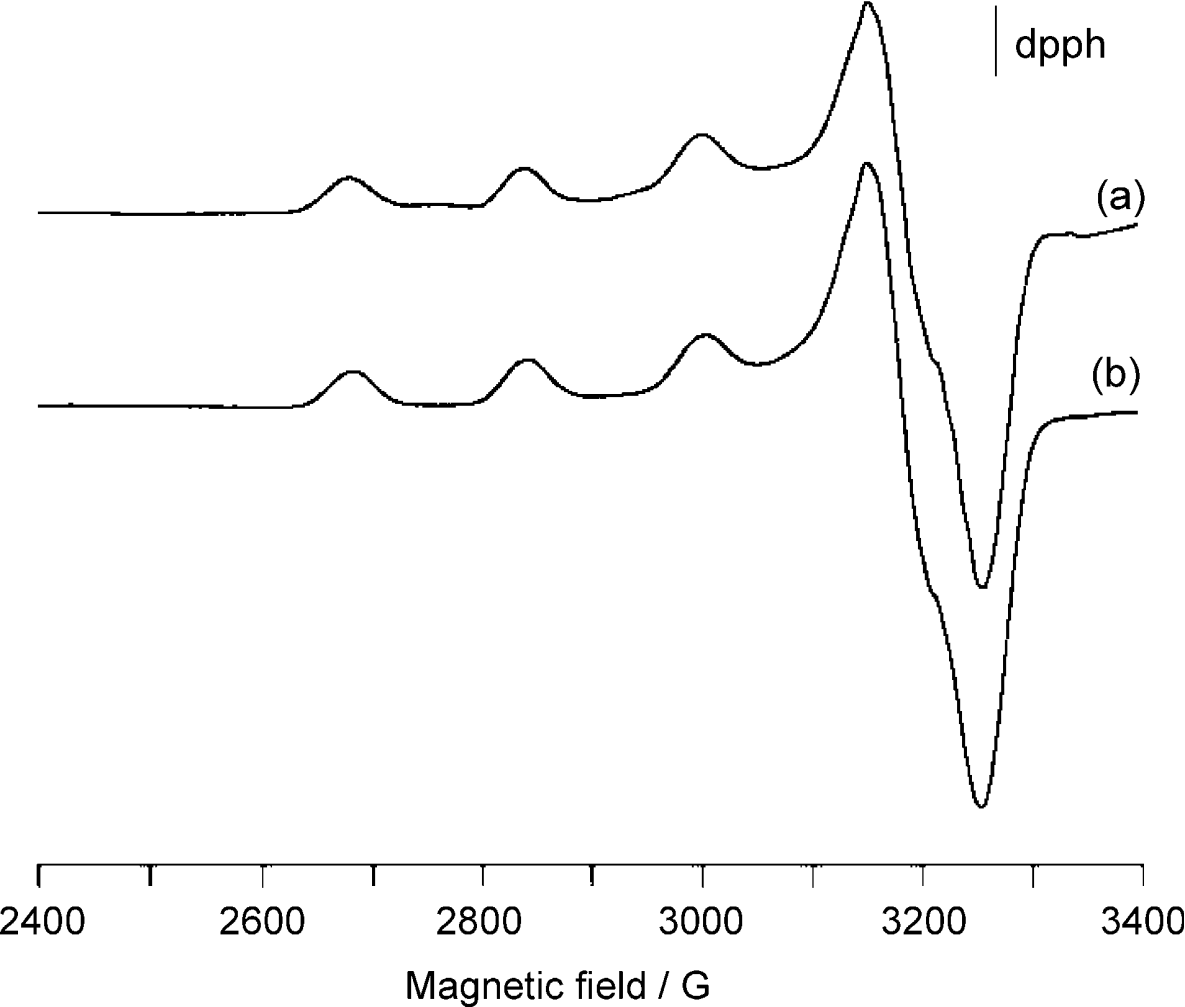
Anisotropic X-band EPR spectra recorded at 100 K of polycrystalline samples of 1 dissolved in a) DMSO and b) DMF.

The solid-state polymeric structure of **1** collapses in acetonitrile, dimethyl sulfoxide (DMSO) or dimethylformamide (DMF), where mononuclear units are formed. The solution EPR spectra in DMSO and DMF are characterised by an axial symmetry with the unpaired electron in the *d*_*x*^2^−*y*^2^_ orbital (Figure 7; for the simulated spectra, see Figures S2 and S3 in the Supporting Information). The parameters are comparable in all of the three solvents, and these similarities can be explained by the breaking of the weak Cu⋅⋅⋅S coordination bond and the formation of discrete [CuL(NCS)] species (acetonitrile: *g*_||_=2.242, *A*_||_= 171×10^−4^ cm^−1^, *g*_⊥_=2.054, *A*_⊥_=15×10^−4^ cm^−1^; DMSO: *g*_||_=2.244, *A*_||_=170×10^−4^ cm^−1^, *g*_⊥_=2.052, *A*_⊥_=14×10^−4^ cm^−1^; and DMF: *g*_||_=2.240, *A*_||_=171×10^−4^ cm^−1^ *g*_⊥_=2.056, *A*_⊥_=14×10^−4^ cm^−1^). The formation of tetragonal mononuclear copper(II) species after the dissolution of the crystalline solid in coordinating organic solvents has been observed for other polymeric NCS-bridged copper(II) compounds.[35]

**Figure 8:**
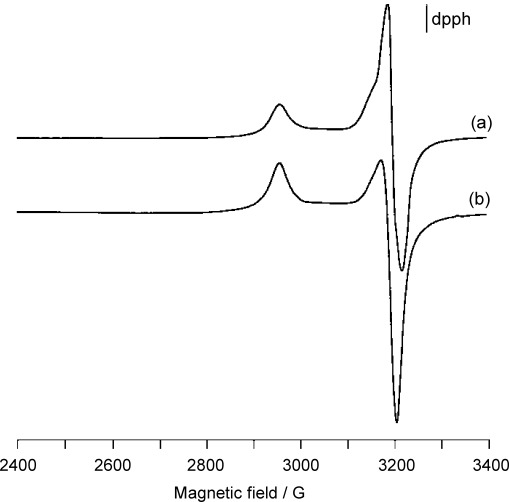
X-band EPR spectra of polycrystalline samples of 2 dissolved in dichloromethane/toluene (50:50 *v*/*v*) at a) RT and b) 100 K.

The powder EPR spectrum of a polycrystalline sample of **2** shows two resonances around 2950 and 3200 G (Figure 8 a). No variation with temperature and no other absorption bands, other than the two at around 2950 and 3200 G, were observed. The values measured are 2.214 for *g*_||_ and 2.039 for *g*_⊥_ at room temperature, and 2.215 for *g*_||_ and 2.042 for *g*_⊥_ at 100 K in dichloromethane/toluene (50:50 *v*/*v*) (Figure 8; for the simulated spectra, see Figures S4 and S5 in the Supporting Information). These values are again consistent with the square-pyramidal geometry of the copper(II) ions in **2** and a *d*_*x*^2^−*y*^2^_ ground state.[30] The experimental behaviour (axial spectrum and no spectral change as a function of the temperature) is in agreement with the data reported in the literature for similar compounds.[36] The forbidden triplet–singlet transition (ΔMS=±2), often detected at half-field for dinuclear copper(II) complexes, is not observed. The *χ*_M_ *T* product of **2** shows a very smooth decrease from approximately 0.85 cm^3^ mol^−1^ K at 300 K down to 0.810 cm^3^ mol^−1^ K at 35 K, which is attributable to the temperature-independent paramagnetism (TIP) of the copper(II) ions (Figure 5). A more abrupt decrease then sets in to reach 0.375 cm^3^ mol^−1^ K at 2 K, clearly indicating the presence of a weak anti-ferromagnetic intramolecular interaction. The experimental data were satisfactorily fitted (solid line in Figure 6) to the expression given in Eq. (1)[37] derived for an exchange-coupled pair of *S*=1/2 spins.


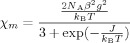
(1)

Adding a fixed TIP value of 1.2×10^−4^ cm^3^ mol^−1^ K resulted in the best-fit parameters *g*=2.10(1) and *J*/*k*_B_=−3.9(1) K (or *J*=2.7(1) cm^−1^). The very strong intensity of the EPR signal is in agreement with the presence of such a weak magnetic interaction and in line with conclusions from a number of magneto–structural correlation studies with dinuclear dichlorido-bridged copper(II) complexes described in the literature.36c, d, g, [38] Indeed, the exchange-coupling constant *J* is expected to depend on the value of the Cu–Cl–Cu bridging angle (*α*), as well as on the bond length of the axial (longer) Cu–Cl bond (*R*), although the different types of arrangement of the two copper polyhedra can also have a great influence on the magnetic behaviour of such complexes.38b–e For a square pyramid sharing one base-to-apex edge, but with parallel basal planes, such as compound **2**, extended Hückel calculations show that the magnetic interaction occurs through a π* interaction between the copper *d*_*x*^2^−*y*^2^_ and the chloride *p* orbitals, and that the extent of the magnetic coupling depends on small structural deviations from the ideal square arrangement of the Cu_2_Cl_2_ core.38e A theoretical correlation between the magnetic coupling and both parameters (*α* and *R*) shows that for small *α* values and relatively short *R* values (**2**: *α*=85.4°, *R*=2.746 Å; Table 2) the magnetic coupling should be very weak;36d in particular, for a value of *θ*/*R* lower than 32.6° Å^−1^ or higher than 34.8° Å^−1^ (31.1° Å^−1^ for **2**) the exchange interaction is anti-ferromagnetic,38b, c which is in agreement with our evaluation of the exchange-coupling constant *J* here.

**Figure 9:**
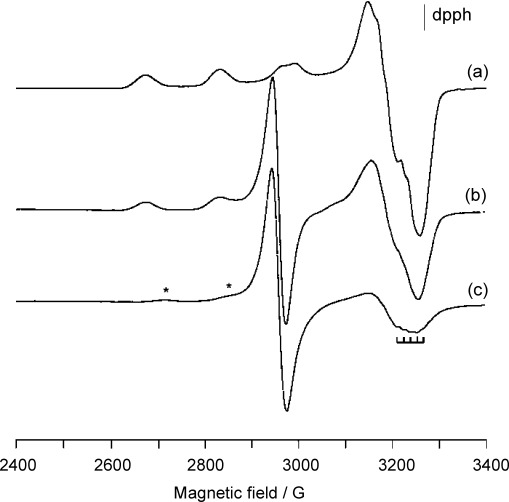
Anisotropic X-band EPR spectra recorded at 100 K of polycrystalline samples of 2 dissolved in a) DMSO, b) DMSO/DMF (50:50 *v*/*v*) and c) DMF. The small graduated line indicates the position of the absorptions due to the super-hyperfine coupling with ^14^N nuclei and the asterisks (*) indicate the parallel resonances of the minor species [CuLCl].

The behaviour of **2** in solution depends on the solvent used (Figure 9). In a non-coordinating solvent (e.g., CH_2_Cl_2_, CHCl_3_, toluene) or in a mixture of non-coordinating solvent (e.g., 50:50 *v*/*v* CH_2_Cl_2_/toluene), the structure is retained (see Figure 8 b), as observed for other similar compounds.[39] On the contrary, in a coordinating solvent (e.g., DMSO or DMF), the longest Cu–Cl bond is broken and mononuclear copper(II) species are formed; these observations are in agreement with what is found in many other similar complexes.36c, e, [40] The EPR spectrum in DMSO shows an axial symmetry and *d*_*x*^2^−*y*^2^_ ground state (Figure 9 a; for the simulated spectrum, see Figure S6 in the Supporting Information). The spectral parameters (*g*_||_=2.251, *A*_||_=168×10^−4^ cm^−1^, *g*_⊥_=2.052, *A*_⊥_=14×10^−4^ cm^−1^), though slightly different from those measured for **1** in DMSO, are consistent with the presence of a chloride rather than a thiocyanato ligand in the fourth equatorial position of the copper ion. Furthermore, the equatorial donor set (N_pyr_, N_imine_, 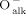, Cl^−^) is confirmed by the *A*_||_ value (168×10^−4^ cm^−1^), which is smaller than that of ([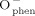, N_imine_, 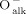, Cl^−^] given by the ligand 2-((*E*)-(2-hydroxyethylimino)methyl)-4-bromophenol (*A*_||_=176.5×10^−4^ cm^−1^);36f however, it is noteworthy that the weak axial coordination of DMSO molecules is also possible. The EPR spectrum in DMF (Figure 9 c; see also Figure S8 in the Supporting Information) is characterised by *g*_⊥_>*g*_||_∼*g*_e_ (*g*_⊥_=2.219, *g*_||_=2.016); this order can be explained by considering a *d*_*z*^2^_ ground state and a trigonal-bipyramidal geometry.[30] It is plausible that DMF inserts into the first coordination sphere of **2**, forming a penta-coordinated species. Interestingly, in DMSO/DMF (50:50 *v*/*v*), both the octahedral [CuLCl(DMSO)_2_] and the trigonal-bipyramidal species [CuLCl(DMF)] are present (Figure 9 b). The binding of a solvent molecule, like DMF, to copper to give a trigonal-bipyramidal complex is observed with the tetradentate calix[6]arene capped by a tris(2-aminoethyl)amine (tren) unit.[41] Remarkably, competitive binding experiments have also demonstrated that, for a coordinating solvent, the preference order for an exchangeable metal site is DMF>ethanol>acetonitrile.[41] The super-hyperfine structure visible in the parallel region of the EPR spectrum (Figure 9 c), which can be attributed to the interaction of the copper(II) unpaired electron with two ^14^N (*I*=1) equivalent nuclei, belonging to the pyridine ring and imino groups. The *A*^N^ value of 15 ×10^−4^ cm^−1^ is in good agreement with those reported in the literature.[42] The weak resonances observable in DMF can be attributed to the minor species [CuLCl(DMF)_2_] with two axially coordinated DMF molecules (Figure S7 in the Supporting Information); the presence of more than one species in an organic solvent, one originating from the dissociation of the dinuclear core, and another originating from their solvation, has already been demonstrated for other di-μ-chloro copper(II) complexes.36e

### Effects on human colorectal carcinoma cells

**Figure 10:**
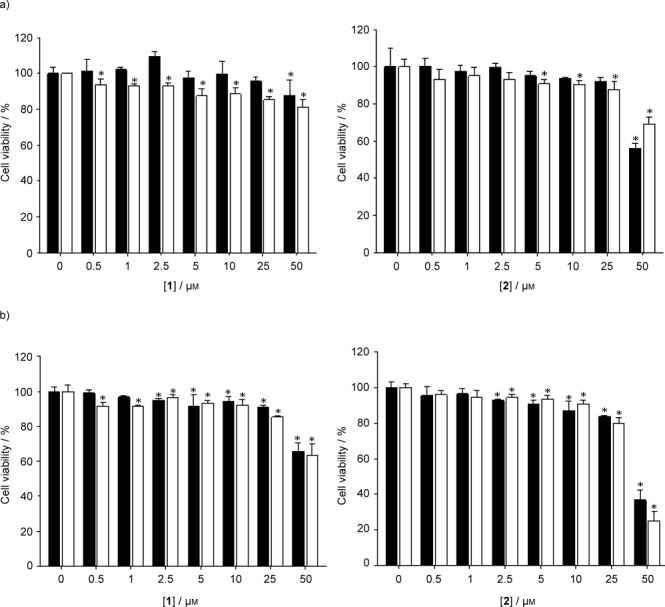
Effects of complexes 1 and 2 on the cell viability of human colorectal carcinoma cells a) COLO-205 and b) HT-29. Cells were treated with 0–50 μm solutions of complexes 1 and 2 for 24 (▪) and 48 h (□). The reported values are the mean ±SD (*n*=3). *p* <0.05 (*) indicates value is significantly different from that of the control.

An uncontrolled cell proliferation is observed in several human diseases including cancer. The potential anticancer activities of both copper compounds have been evaluated using a model system for the in vitro control of tumor-cell proliferation. Figure 10 illustrates the effects of compounds **1** and **2** on the cell-population growth of human colorectal carcinoma cells (COLO-205 and HT-29). The data indicate that an addition of **1** and **2** to the growth medium gives rise to a decrease in the cell-population growth of COLO-205 and HT-29 cells. In addition, the results from an MTT assay with the HT-29 human colorectal carcinoma cell line reveal that **2** has the higher inhibitory activity of the two compounds. The programmed cell death can be activated through two main pathways, namely the mitochondrion-dependent pathway (the intrinsic pathway) and the death receptor-dependent pathway (the extrinsic or Fas-mediated pathway).[43] Hence, investigations regarding the apoptotic properties of **2** on HT-29 human colorectal carcinoma cells suggest that both the Fas- and mitochondria-mediated pathways are induced by this compound.

## Conclusion

In the present study, two copper(II) complexes from a hydrazone-based ligand have been prepared and fully characterised. Magnetic studies (EPR and variable-temperature magnetic susceptibility measurements) show that the respective bridging anions, that is, thiocyanate (**1**) and chloride (**2**), generate weak exchange-coupling interactions between the metal centres, which is in agreement with literature data for related polynuclear copper(II) systems exhibiting analogous structural features. Interestingly, noticeable biological properties regarding inhibitory effects on cell-population growth in human colorectal carcinoma cells have been observed with these complexes (particularly with **2**), validating further the high potential of hydrazone-type ligands to develop new antitumor agents.

## Experimental Section

### Chemistry

**Physical techniques**: Elemental analyses were performed on a CHN-OS rapid elemental analyser (Heraeus, Hanau, Germany) at the Instrument Centre of the National Chung Hsing University (NCHU). IR spectra were recorded in the range of 4000–200 cm^−1^ as KBr pellets on an 883-IR spectrophotometer (PerkinElmer, Waltham, MA, USA). Electronic spectra were measured in MeOH on an U 3400 UV-Vis-NIR spectrophotometer (Hitachi, Japan, Tokyo). Magnetic susceptibility measurements were carried out with a SQUID MPMS-XL susceptometer apparatus (Quantum Design, San Diego, CA, USA) working in the temperature range of 2–300 K, under a magnetic field of approx. 0.1 *T* (2–30 K) and 1 *T* (35–300 K). Electron paramagnetic resonance (EPR) spectra were recorded from 0 to 10 000 G at RT or at 100 K with an X-band (9.15 GHz) E-9 spectrometer (Varian, Palo Alto, CA, USA). The EPR parameters reported in the text were obtained by simulating the spectra with the software WinEPR SimFonia (Bruker, Billerca, MA, USA).[44] In all the simulations, second-order effects were taken into account, and the Lorentzian–Gaussian ratio, affecting the line shape, was set to 1.

**Reagents**: Acetylhydrazide (Acros Organics, Geel, Belgium), 2-acetylpyridine (Alfa Aesar, Ward Hill, MA, USA), Cu(NO_3_)_2_⋅3H_2_O (Hayashi, Kyoto, Japan), anhyd CuCl_2_ and NaSCN (Showa Chemicals, Tokyo, Japan) were used as received without further purification. All solvents used were of reagent grade.

***N*****′-[1-(pyridin-2-yl)ethylidene]acetohydrazide (HL)**: As described previously,[17] acetylhydrazide (0.074 g, 1.0 mmol) was reacted with 2-acetylpyridine (0.112 mL, 1.0 mmol) in MeOH (15 mL). After 2 h at reflux, the pale yellow methanolic salt was cooled to RT. The solvent was removed in vacuo, and the Schiff-base ligand was obtained as a light-yellow liquid, which was used without further purification.

**[CuL(NCS)]**_***n***_ **(1)**: A solution of Cu(NO_3_)_2_⋅5H_2_O (0.241 g, 1 mmol) in MeOH (20 mL) was treated with HL (0.177 g, 1 mmol) instantly giving a green solution. Subsequently, a solution of NaSCN (0.081 g, 1 mmol) in a minimum volume of H_2_O was added, and the solution was stirred for 10 min at RT. The reaction mixture was then heated to reflux for 15 min. After cooling to RT, the solution was left unperturbed for 3 d, and the resultant crystals were isolated by filtration, washed with H_2_O and dried in air to give dark-green, rectangular-shaped, single crystals of **1** (0.184 g, 62 %): Anal. calcd for C_10_H_10_CuN_4_OS: C 40.33, H 3.38, N 18.81, found: C 40.37, H 3.42, N 18.77.

**[Cu_2_L_2_Cl_2_] (2)**: A solution of CuCl_2_ (0.134 g, 1 mmol) in hot MeOH (25 mL) was treated with HL (0.177 g, 1 mmol) and stirred at 40 °C for 30 min. After cooling to RT, the solution was left unperturbed for 3 d at 4°C, and the resultant crystals were isolated by filtration and air-dried to give dark-green, square-shaped, single crystals of **2** (0.368 g, 67 %): Anal. calcd for C_18_H_20_Cl_2_Cu_2_N_6_O_2_: C 39.28, H 3.66, N 15.27, found: C 39.41, H 3.59, N 15.39.

### Biology

*Cell culture*: Human colorectal carcinoma cells (COLO-205 and HT-29) were provided by Dr. Min Hsiung Pan (National Kaohsiung Marine University, Kaohsiung, Taiwan). COLO-205 and HT-29 cells were grown in 90 % RPMI 1640 medium supplemented with fetal bovine serum (10 %), penicillin (100 U mL^−1^), and streptomycin (100 μg mL^−1^). COLO-205 and HT-29 cells were cultured at 37 °C in a 5 % CO_2_ humidified atmosphere.

*MTT assay*: The 3-(4,5-dimethylthiazol-2-yl)-2,5-diphenyl tetrazolium bromide (MTT) assay (Sigma Chemical Co., St. Louis, MO, USA) was performed according to the method of Mosmann.[45] COLO-205 or HT-29 cells were plated into 96-well microtiter plates at a density of 1×10^4^ cells per well. After 24 h, the culture medium was replaced by aliquots (200 μL) of compounds **1** or **2** in DMSO (0–50 μm), and the cells were incubated for 24 and 48 h. The final concentration of DMSO was less than 0.1 % in cell culture medium. The culture medium was removed and replaced by fresh culture medium (90 μL). Sterile filtered MTT solution (10 µL, 5 mg mL^−1^) in phosphate-buffered saline (PBS, pH 7.4) were added to each well, thereby reaching a final MTT concentration of 0.5 mg mL^−1^. After 5 h, unreacted dye was removed, and the insoluble formazan crystals were dissolved in DMSO (200 µL per well) and measured spectrophotometrically in a VersaMax tunable microplate reader (Molecular Devices, Sunnyvale, CA, USA) at 570 nm. The cell viability (%) relative to control wells containing cell culture medium without samples was calculated using Eq. (2). Experiments were performed in triplicate, and data represent the mean value.


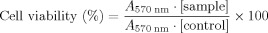
(2)

### X-Ray crystallography

Details concerning crystal data, data collection characteristics and structure refinement are summarised in Table 1. Single crystals of **1** and **2** were mounted separately on capillaries and transferred to a goniostat. Diffraction data were collected at 298(2) K for **1**, and 150(2) K for **2**, under a nitrogen stream, on a SMART CCD diffractometer (Bruker, Billerica, MA, USA) with graphite-monochromated Mo-Kα radiation. The *ω*:2*θ* scan technique was applied within a *θ* range of 2.13–28.78° for **1**, and 2.53–28.73° for **2**. No significant crystal decay was observed. Data were corrected for absorption empirically by means of ψ scans. A total of 17 434 (**1**) and 7940 (**2**) reflections were collected, from which 3056 (**1**) and 2426 (**2**) independent [*R*(int)=0.0231 and 0.0453, respectively] reflections were measured. The stability of the crystals was checked by measuring standard reflections at fixed intervals during the data collection. However, no significant loss of intensity was noted. Data were processed using the software CrysAlis CCD and CrysAlis RED (Oxford Diffraction Ltd, UK).[46] The structures were solved by direct methods using the SHELXTL PLUS[47] system and refined using all data by a full-matrix, least-squares method based on *F*^2^ using SHELXL93.[48] The functions minimised were Σ*w*[|*F*o|^2^−[|*F*c|^2^]^2^, where *w*=[*σ*^2^(*I*)+(0.0849*P*)^2^+0.3606*P*]^−1^ for **1**, and *w*=[*σ*^2^(*I*)+(0.0439*P*)^2^+0.5656*P*]^−1^ for **2**, with *P*=(|*F*o|^2^+2|*F*c|^2^)/3. The hydrogen atom positions were calculated, and the hydrogen atoms were constrained to idealised geometries and treated as riding, where the hydrogen atom displacement parameter was calculated from the equivalent isotropic displacement parameter of the bound atom. CCDC-793717 and CCDC-821330 contain the crystallographic data for **1** and **2**, respectively. These data can be obtained free of charge from the Cambridge Crystallographic Data Centre via http://www.ccdc.cam.ac.uk.

## Supplementary material

Detailed facts of importance to specialist readers are published as ”Supporting Information”. Such documents are peer-reviewed, but not copy-edited or typeset. They are made available as submitted by the authors.
