# Supplementary material for: End-to-End Thiocyanato-Bridged Helical Chain Polymer and Dichlorido-Bridged Copper(II) Complexes with a Hydrazone Ligand: Synthesis, Characterisation by Electron Paramagnetic Resonance and Variable-Temperature Magnetic Studies, and Inhibitory Effects on Human Colorectal Carcinoma Cells
Source: ChemistryOpen. 2012 Mar 13;1(2):80–9. doi: 10.1002/open.201100011 (PMC3922458; doi:10.1002/open.201100011)
Supplement: Supplementary file 1 [file open0001-0080-SD1.pdf]

ChemistryOPEN

Supporting Information

© Copyright Wiley-VCH Verlag GmbH & Co. KGaA, 69451 Weinheim, 2012

End-to-End Thiocyanato-Bridged Helical Chain Polymer and Dichlorido-Bridged Copper(II) Complexes with a Hydrazone Ligand: Synthesis, Characterisation by Electron Paramagnetic Resonance and Variable-Temperature Magnetic Studies, and Inhibitory Effects on Human Colorectal Carcinoma Cells

Kuheli Das,[a] Amitabha Datta,*[b] Chittaranjan Sinha,[a] Jui-Hsien Huang,*[b]
Eugenio Garribba,[c] Ching-Sheng Hsiao,[b] and Chin-Lin Hsu[d]

open_201100011_sm_miscellaneous_information.pdf

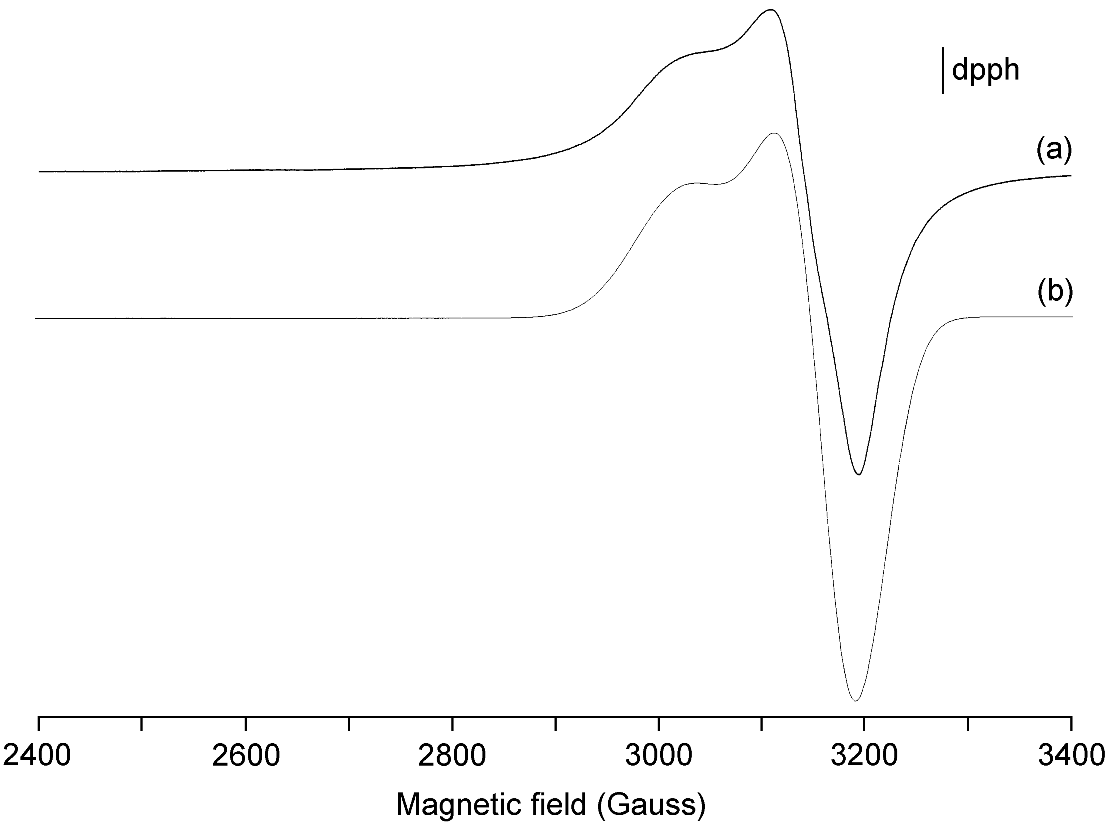

Figure S1. X-band EPR spectrum of polycrystalline sample of **1** at 100 K: (a) experimental and (b) simulated spectrum. The spectrum was simulated with g_x = 2.172, g_y = 2.078, g_z = 2.050.

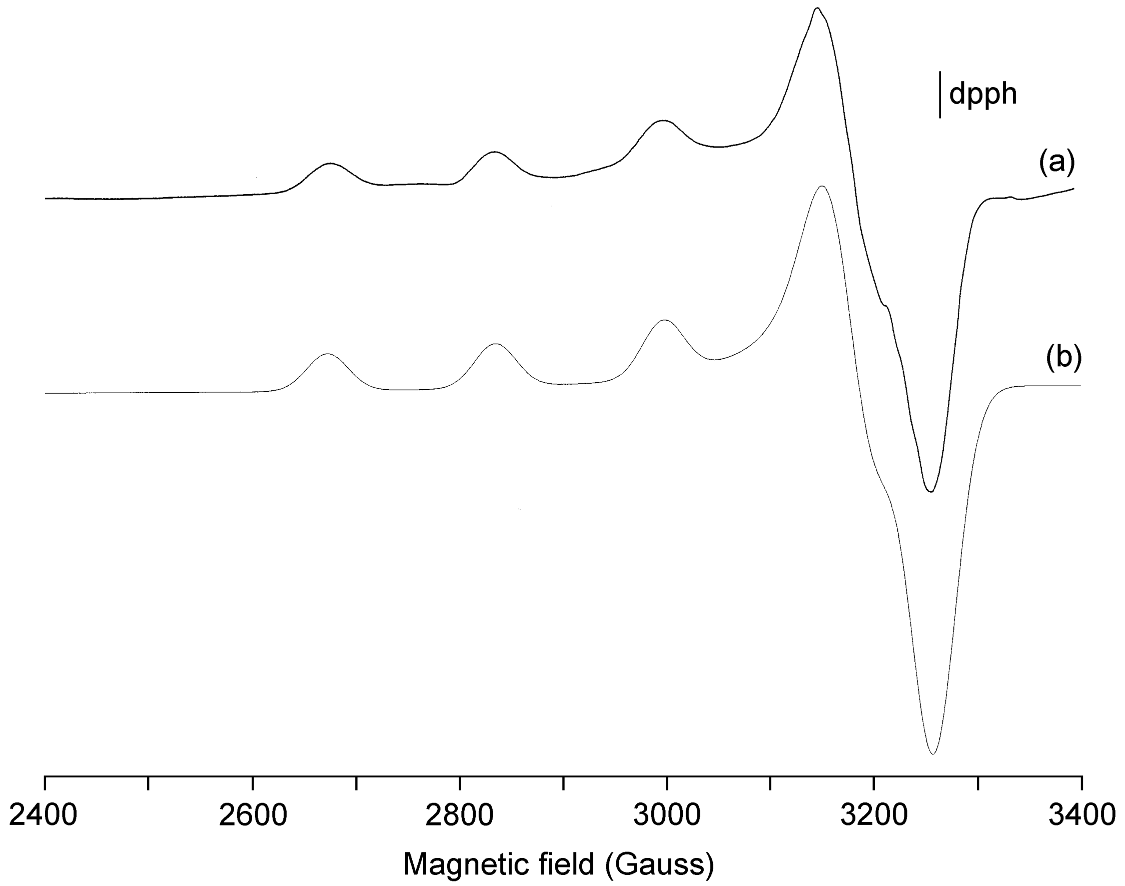

Figure S2. X-band EPR spectrum of polycrystalline sample of **1** dissolved in DMSO at 100 K: (a) experimental and (b) simulated spectrum. The spectrum was simulated with $g_{\parallel}$ = 2.244, $A_{\parallel}$ = 170 × 10^{-4} cm^{-1}, $g_{\perp}$ = 2.052, $A_{\perp}$ = 14 × 10^{-4} cm^{-1}.

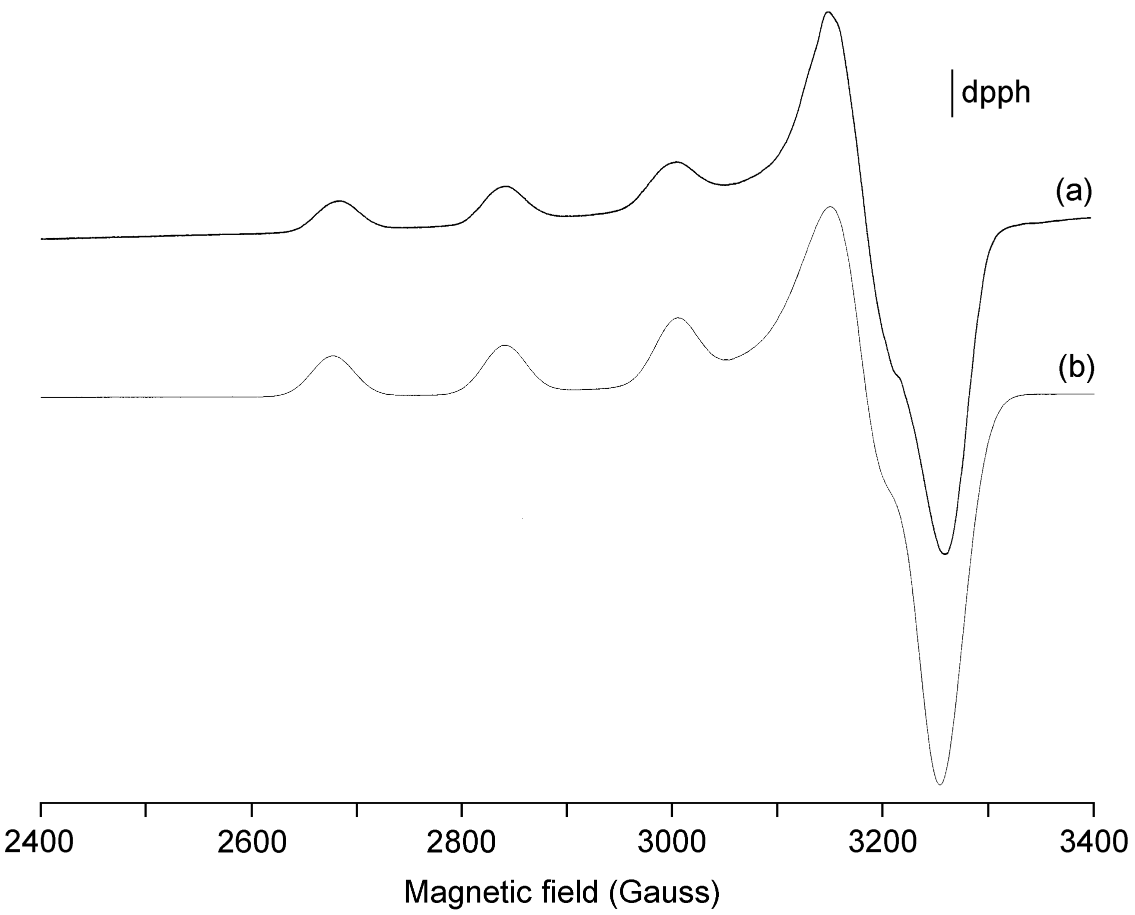

Figure S3. X-band EPR spectrum of polycrystalline sample of **1** dissolved in DMF at 100 K: (a) experimental and (b) simulated spectrum. The spectrum was simulated with $g_{\|} = 2.240$, $A_{\|} = 171 \times 10^{-4}$ cm^{-1} $g_{\perp} = 2.056$, $A_{\perp} = 14 \times 10^{-4}$ cm^{-1}.

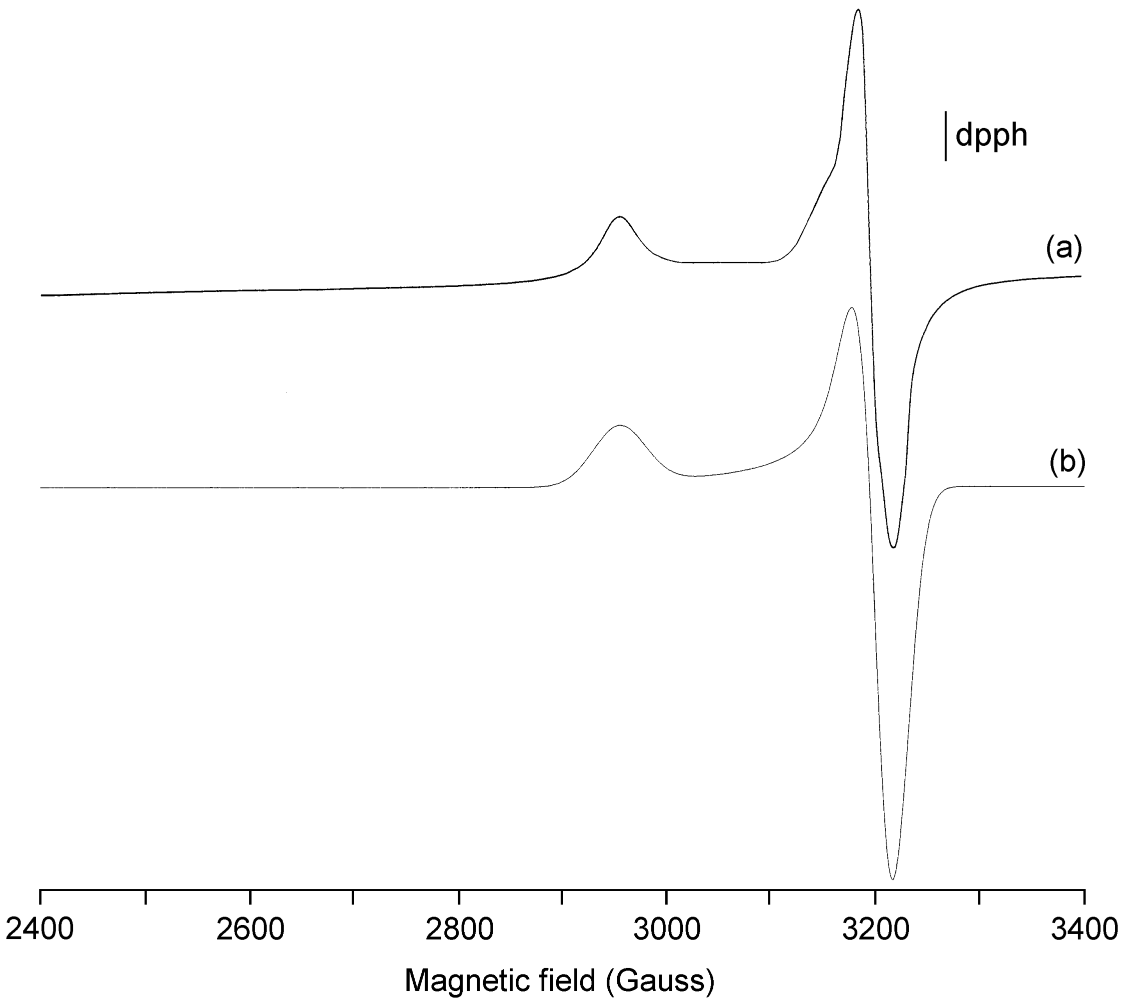

Figure S4. X-band EPR spectrum of polycrystalline sample of **2** at RT: (a) experimental and (b) simulated spectrum. The spectrum was simulated with $g_{\|} = 2.2140$, $g_{\perp} = 2.039$.

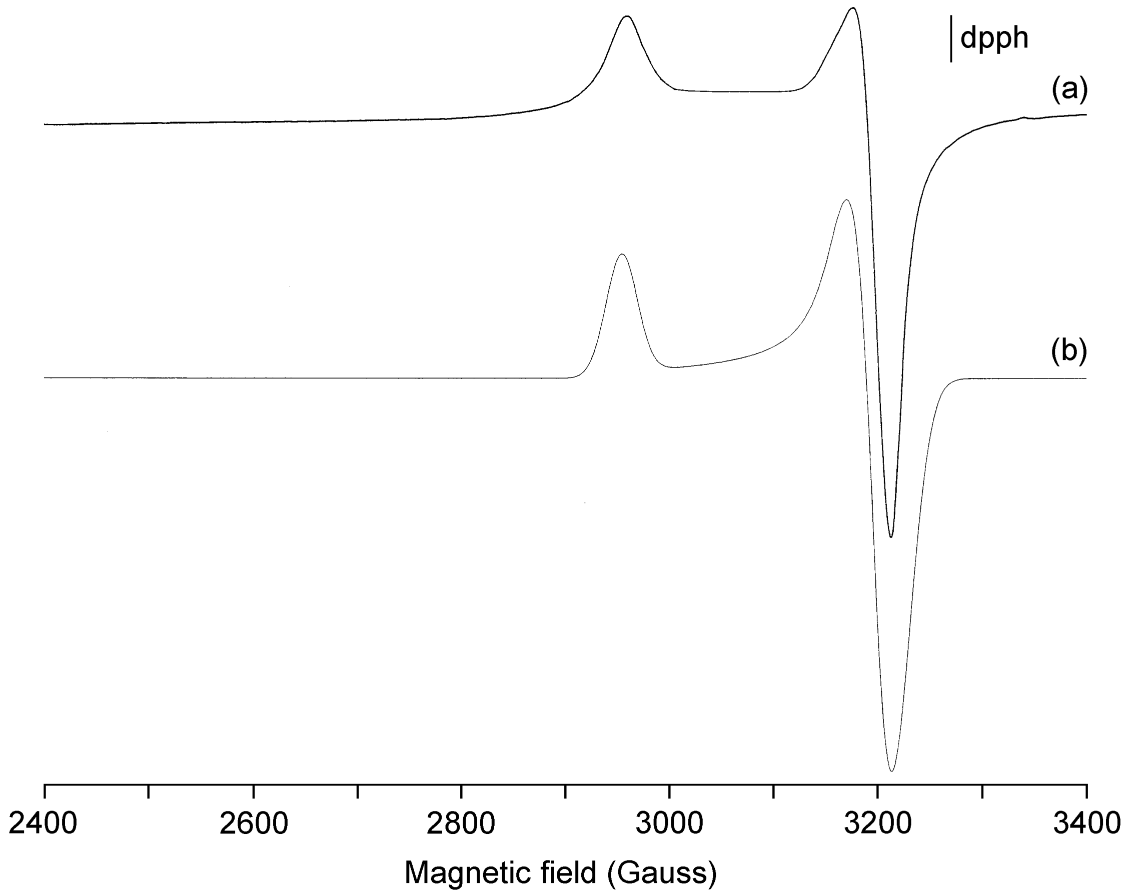

Figure S5. X-band EPR spectrum of polycrystalline sample of **2** dissolved in a mixture CH_2Cl_2/toluene 50:50 v/v: (a) experimental and (b) simulated spectrum. The spectrum was simulated with $g_{||}$ = 2.215, $g_{\perp}$ = 2.042.

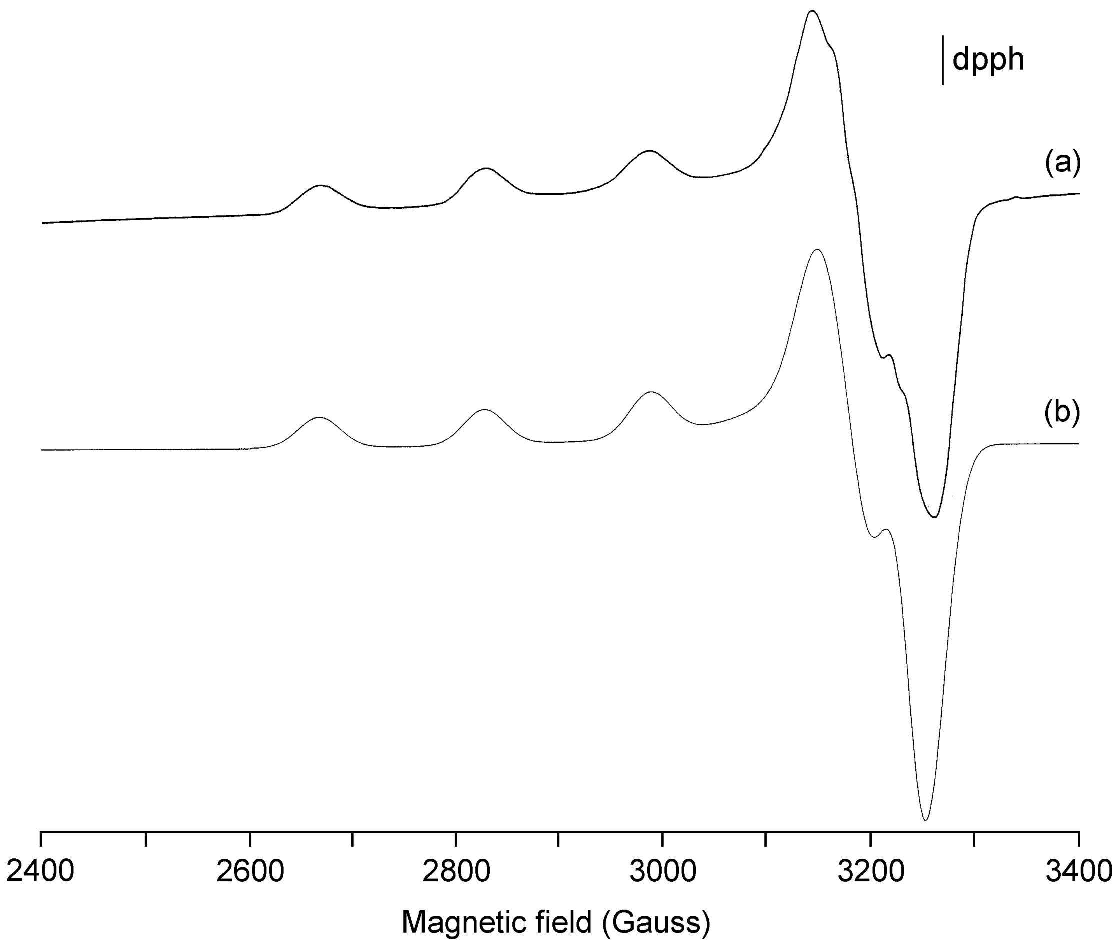

Figure S6. X-band EPR spectrum of polycrystalline sample of **2** dissolved in DMSO at 100 K: (a) experimental and (b) simulated spectrum. The spectrum was simulated with $g_{||}$ = 2.251, $A_{||}$ = 168 × 10^{-4} cm^{-1}, $g_{\perp}$ = 2.052, $A_{\perp}$ = 14 × 10^{-4} cm^{-1}. It is attributed to the species [Cu**L**Cl$(DMSO)_2$].

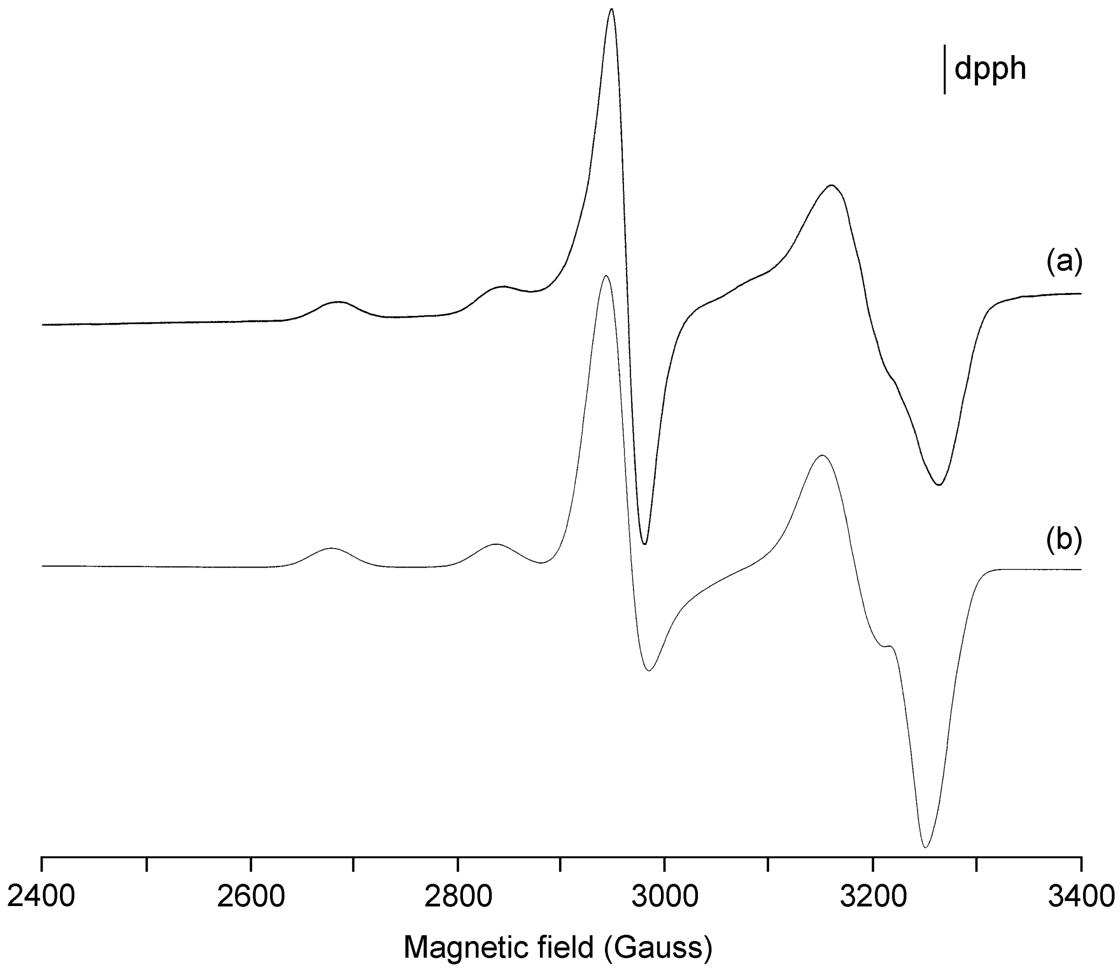

Figure S7. X-band EPR spectrum of polycrystalline sample of **2** dissolved in a mixture DMSO/DMF 50:50 v/v at 100 K: (a) experimental and (b) simulated spectrum. The spectrum was simulated considering a percentage amount of [Cu**L**Cl(DMF)] of 55% and of [Cu**L**Cl(DMSO)$_2$] (see Figure S6) of 45%. The EPR parameters of [Cu**L**Cl(DMF)] are $g_\perp$ = 2.219, $g_{||}$ = 2.019, A^N = 15 × 10^{-4} cm^{-1}, and of [Cu**L**Cl(DMSO)$_2$] are $g_{||}$ = 2.251, $A_{||}$ = 168 × 10^{-4} cm^{-1}, $g_\perp$ = 2.052, $A_\perp$ = 14 × 10^{-4} cm^{-1} (see Fig. S6).

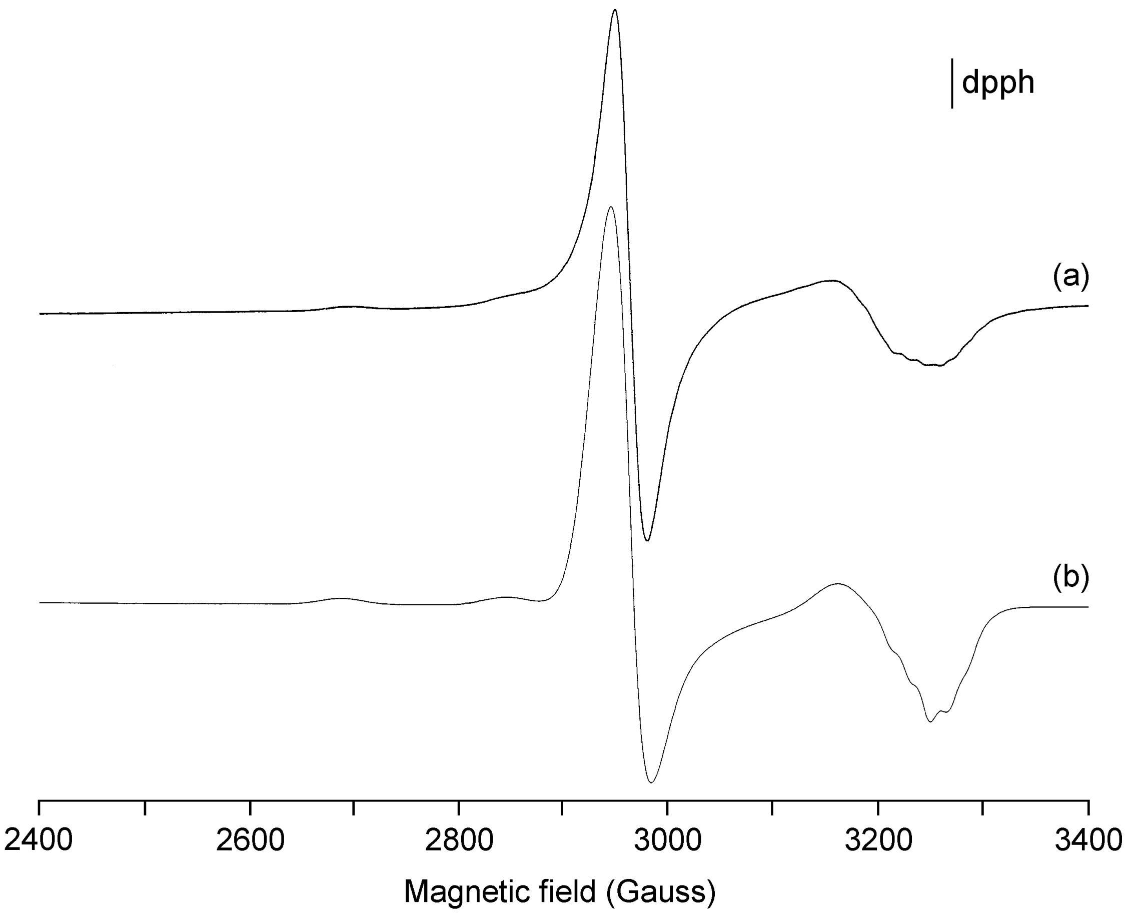

Figure S8. X-band EPR spectrum of polycrystalline sample of **2** dissolved in DMF at 100 K: (a) experimental and (b) simulated spectrum. The spectrum was simulated considering a percentage amount of [Cu**L**Cl(DMF)] of 90% and of [Cu**L**Cl(DMF)$_2$] of 10%. The EPR parameters of [Cu**L**Cl(DMF)] are $g_{\perp}$ = 2.219, $g_{||}$ = 2.019, A^{N} = 15 × 10^{-4} cm^{-1}, and of [Cu**L**Cl(DMF)$_2$] are $g_{||}$ = 2.240, $A_{||}$ = 165 × 10^{-4} cm^{-1}, $g_{\perp}$ = 2.044, $A_{\perp}$ = 14 × 10^{-4} cm^{-1}.
